# The lifetime cost to English students of borrowing to invest in a medical degree: a gender comparison using data from the Office for National Statistics

**DOI:** 10.1136/bmjopen-2014-007335

**Published:** 2015-04-01

**Authors:** Marco G Ercolani, Ravinder S Vohra, Fiona Carmichael, Karanjit Mangat, Derek Alderson

**Affiliations:** 1 Birmingham Business School, University of Birmingham, Birmingham, UK; 2 Academic Department of Surgery, University of Birmingham, Birmingham, UK; 3 The Queen Elizabeth Hospital Birmingham, Birmingham, UK

**Keywords:** MEDICAL EDUCATION & TRAINING, JOURNALISM (see Medical Journalism)

## Abstract

**Objective:**

To evaluate this impact on male and female English medical graduates by estimating the total time and amount repaid on loans taken out with the UK's Student Loans Company (SLC).

**Setting:**

UK.

**Participants:**

4286 respondents with a medical degree in the Labour Force Surveys administered by the Office for National Statistics (ONS) between 1997 and 2014.

**Outcomes:**

Age-salary profiles were generated to estimate the repayment profiles for different levels of initial graduate debt.

**Results:**

2195 female and 2149 male medical graduates were interviewed by the ONS. Those working full-time (73.1% females and 96.1% males) were analysed in greater depth. Following standardisation to 2014 prices, average full-time male graduates earned up to 35% more than females by the age of 55. The initial graduate debt from tuition fees alone amounts to £39 945.69. Owing to interest charges on this debt the average full-time male graduate repays £57 303 over 20 years, while the average female earns less and so repays £61 809 over 26 years. When additional SLC loans are required for maintenance, the initial graduate debt can be as high as £81 916 and, as SLC debt is written off 30 years after graduation, the average female repays £75 786 while the average male repays £110 644.

**Conclusions:**

Medical graduates on an average salary are unlikely to repay their SLC debt in full. This is a consequence of higher university fees and as SLC debt is written off 30 years after graduation. This results in the average female graduate repaying more when debt is low, but a lower amount when debt is high compared to male graduates.

**Strengths and limitations of this study**

- This study reports the lifetime cost to medical graduates of repaying the Student Loans Company under the new £9000 annual fees regime.
- Once loans for living costs are factored in, medical graduates are unlikely to repay their debt in full before it is written off at the end of the 30th year after graduation.
- If the amount borrowed is more than about £50 000, someone on an average female medical graduate salary will typically repay less than her male counterpart because she earns less and repayments are mainly based on the level of earnings.
- However, if the amount borrowed is less than about £50 000, those on the average female graduate salary repay more than their male counterparts as they accrue more interest charges.
- The analysis in this study is based on the assumption that the pay scales of medical graduates will rise in line with price inflation and that there will be no changes to the terms of the student loans. Small changes in either of these two sets of assumptions could have huge implications for medical graduates’ actual lifetime repayments over the next 30 years.

## Introduction

Over the past decade, annual tuition fees for English students attending UK universities have risen from £1000 when introduced in 1998 to £9000 currently.^1^ This is mirrored by a rise in graduate debt, which is particularly important for courses that involve prolonged periods of study such as medicine. The British Medical Association (BMA) has suggested that graduate debt for current medical students could be in excess of £70 000.^2^ Such levels of graduate debt may affect the recruitment of students to a career in medicine in the UK as some may choose alternative careers and others may decide to study medicine abroad.

The Student Loans Company (SLC), under its ‘Plan 2’ scheme introduced in September 2012, changed both the amounts that it will lend to students and the rules governing the repayment of the resulting debt. The SLC will lend students £9000 *per annum* (*pa*) for tuition alone plus a separate maintenance loan^3^ to cover students’ housing costs and food. Means tested grants and bursaries for final year tuition fees are available for selected students.^4^ Currently, SLC debts are automatically written off 30 years after graduating, regardless of the remaining debt. Repayments are based on 9% of salary above £21 000 (at 2016 prices under the current SLC Plan 2) rather than the amount of debt. Early research^5^ ^6^ has analysed the implications of the SLC Plan 2 scheme, but was limited to those on average graduate salaries and 3-year degrees. However, to date, there has been no detailed analysis of the implications for the prolonged periods of study and higher debt for courses such as medicine.

To understand how much a medical graduate might repay, an accurate estimation of gross graduate salaries is required. For medical graduates, pay scales^7^ do not reflect the actual salaries earned as these can depend on additional work hours, sessions worked, flexible working and career breaks. In addition, there are notable differences between the salaries of male and female medical graduates.^8^ ^9^

To overcome these issues, a representative data set of medical graduates’ actual salaries is required. The Labour Force Survey (LFS) is the largest survey undertaken by the Office for National Statistics (ONS) that records household salaries. The LFS collects data continuously on 1% of the UK population each year and provides official measures on employment rates.^10^ To ensure an even coverage of the UK population and minimise sources of sampling bias, the ONS samples households randomly using the Royal Mail’s Postcode Address File. Despite the lengthy questionnaire format, the ONS conducts interviews in person or over the telephone to maximise response rates.^11^

In this study, the LFS was used to calculate salary profiles for the average medical graduate. Using this and the repayment schedules from the SLC, a model for the repayment of graduate debt for English students studying in the UK was developed. Total amounts repaid over the lifetime of these SLC loans were calculated based on different amounts of graduate debt.

## Methods

### Male and female medical graduates’ salaries following graduation

LFS data between April 1997 and June 2014 were used^10^ to obtain a substantial sample of medical graduates on which to base our calculations of average earnings. Their salaries were then grouped by age and gender.

This sample of medical graduates included those who may or may not be practising clinicians or who work in or outside hospital settings. The main analysis considered full-time workers alone, defined as those working more than 30 hours per week,^12^ while a supplementary analysis included both part-time and full-time workers. Gross salaries and other monetary values were re-indexed to 2014 prices using the retail prices index (code CHAW) from the ONS.^13^ Caution must be exercised when collecting data over such a long time period because average real salaries change over time, but this was necessary to achieve a reasonably large sample size. Fortunately, from a research standpoint, medical salaries have risen almost in line with Retail Price Inflation (RPI) so that they have hardly risen in real terms (see online supplementary figure S1). It was therefore assumed that future salaries will remain steady in real terms.

### Debt from fees for a medical degree

It is assumed here that a medical degree takes 5 years to complete and that most Universities will charge the maximum allowable £9000 *pa* for tuition fees. It should be realised that this is currently only true for English students who study in the UK. Tuition fees vary within the UK by country of study and student's country of origin. For example, Scottish students studying in Scotland have most of their £9000 fees paid by Student Awards Agency for Scotland and there are varying maximum amounts both Welsh and Northern Irish students are expected to pay.

The BMA and the National Health Service (NHS) provide a bursary for the final year's fees, so this was discounted. Repayment calculations were based on the SLC ‘Plan 2’ scheme that allows loans up to £9000 *pa* with annual interest at a rate of 3% plus RPI. These interest charges begin when the loan starts and continue thereafter. Therefore, a medical graduate who borrows to cover all tuition fees under the current ‘Plan 2’ system, at 2014 prices, would graduate with a debt of £39 946 (see equation 1 in the online supplementary file).

### Debt from maintenance loans for a medical degree

The SLC ‘Plan 2’ also offers maintenance loans of £4375 for students living at home; £7675 for students living in London; and £5500 for students living at universities elsewhere in the UK. Total maintenance debt for a student living at home was therefore calculated as £23 924, for a student living away from home and outside of London as £30 076, and for a student who studies in London and away from home as £41 970 (see equations 2–4 in the online supplementary file).

### Repayment of fees (for any level of debt)

Under the terms of the SLC ‘Plan 2’, repayments are based on graduate gross income, not the level of debt. As *per* these rules, the Pension Contribution Rate (PCR) was subtracted from the gross salary (see online supplementary table S1^14^ ^15^). Models of the yearly repayment schedules were created (see equation 5 in the online supplementary file). This analysis assumed that repayments are made each year rather than each month. It was also assumed that interest is charged at the same rate (3% plus RPI) as is during the degree and for salaries above £41 000, given that medical salaries frequently exceed this. The formula used to calculate the running graduate debt at the end of each year is equation (6) in the online supplementary file.

### Analyses

These were conducted using StataCorp Stata Statistical Software (Release V.13. 2013 College Station, Texas, USA: StataCorp LP^16^). The report was written in accordance with the Strengthening the Reporting of Observational Studies in Epidemiology (STROBE) guidelines.

## Results

### Sample demographics

The overall LFS response rates varied between 75% and 50% during the period studied.^11^ There were 2195 female and 2149 male medical graduates identified in the LFS. The proportion of female medical graduates increased from 39.3% to 56.0% over the period despite the female population within the LFS remaining constant at 51.5%. General demographics are shown in table 1. There were significant gender differences in age groups, proportions in hospital practice and numbers working full time. For the rest of the reported analysis, only full-time workers were included. This equated to 1589 females and 2029 males. For this group of full-time workers, there was a greater proportion of females in the 23–29 age range (59.4%) and a greater proportion of males in the 40–64 age range (63.4%). Furthermore, 73.6% of males worked in a hospital setting compared to 66.7% of females.

**Table 1:** Gender and age composition of LFS respondents with a medical degree: April 1997–June 2014

	Male	Female	Test for difference in gender proportions	
			χ^2^ test	p Value
Age range				
23–29	327 (15)	519 (24)	39.6	<0.001
30–39	734 (34)	791 (36)	1.1	0.294
40–64	1088 (51)	885 (40)	25.4	<0.001
* Total*	*2149 (100%)*	*2195 (100%)*	*66.1*	*<0.001*
Place of work				
Hospital	1565 (73)	1333 (61)	23.8	<0.001
Non-hospital	584 (27)	862 (39)	47.7	<0.001
* Total*	*2149 (100%)*	*2195 (100%)*	*71.5*	*<0.001*
Type of work				
Part-time	82 (4)	586 (27)	366.4	<0.001
Full-time	2029 (96)	1589 (73)	67.5	<0.001
* Total*	*2111 (100%)*	*2175 (100%)*	*432.9*	*<0.001*

			t test	p Value

*Average weekly* *hours among* *full-time workers*	*£48.95* [11]	*£44.50* [10]	12.5	<0.001
*Average hourly* *wage among* *full-time workers*	*£30.74* [15]	*£24.48* [12]	13.5	<0.001

Values in parentheses () are column percentages.

Values in square brackets [ ] are standard deviations.

LFS, Labour Force Survey.

### Age-salary profiles

**Figure 1:**
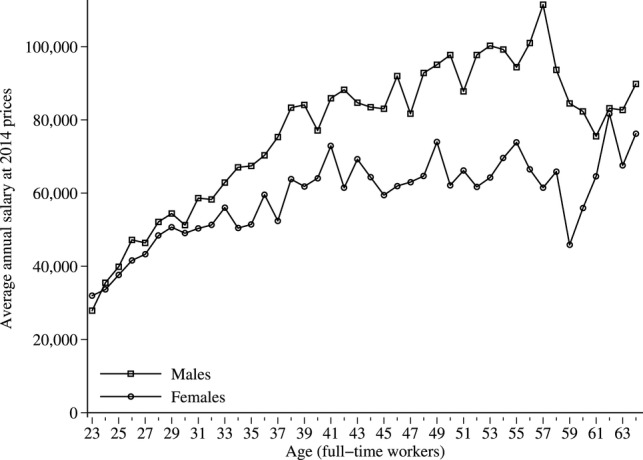
Average salaries for medical graduates working full-time (over 30 basic hours per week).

Average full-time salaries increased with age and then gradually decreased after the age of 55 (figure 1). Though female and male graduate salaries were initially similar, they gradually diverged such that at the age of 55, males earned 35% more than females. The gender difference in annual earnings is mainly due to differences in hourly wage rates rather than number of hours worked (table 1).

### Graduate debt and repayments

**Figure 2:**
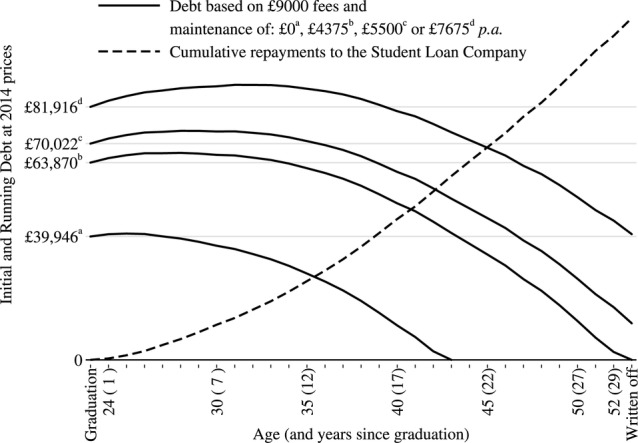
Average debt repayment profiles for full-time male medical graduates.

**Figure 3:**
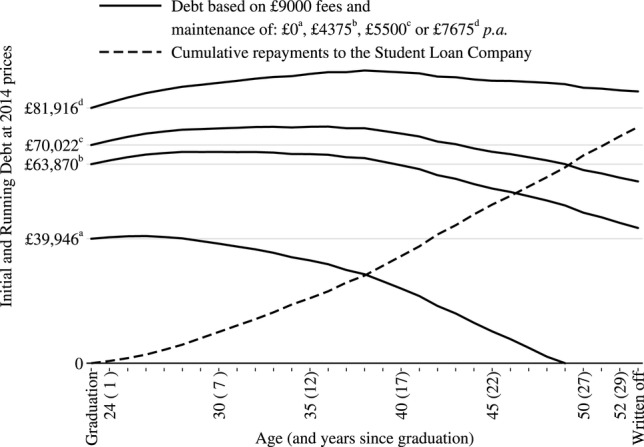
Average debt repayment profiles for full-time female medical graduates.

Using the resulting average age-salary profiles, projected future repayments and cumulative debt for male and female medical graduates who work full-time were generated, as shown in figures 2 and 3 (see online supplementary tables S2 and S3). Considering the repayments of those who borrowed £39 945.69 for tuition fees alone, full-time male graduates would repay a total of £57 303 and clear their debt over 20 years. Full-time female graduates would repay a total of £61 809 and clear their debt over 26 years for the same initial debt as males.

Calculation of future repayments for full-time medical graduates who also required loans for maintenance costs showed that the yearly repayments and the total repaid over 30 years will remain the same, regardless of the level of debt at graduation (figures 2 and 3 and online supplementary tables S2 and S3), with the caveat that the average male graduate who borrows £4375 p.a. for maintenance may repay all his debt in the 30th year of work. The total repaid, however, varies by gender with the average female repaying less than the average male. This is linked to a lower average female medical graduate salary.

**Figure 4:**
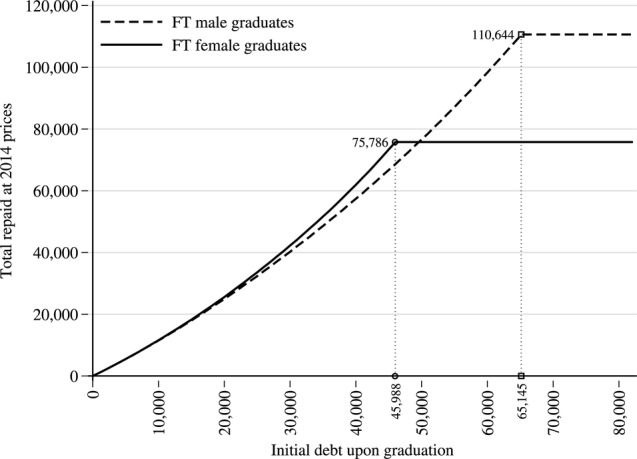
Initial debt to total repayment profiles for full-time medical graduates.

For full-time medical graduates, figure 4 illustrates the total repaid for any level of initial debt. The total repaid increases with the initial level of debt, but only up to a certain level as any remaining debt is written off after 30 years. The repayment profiles for full-time female and male medical graduates are different because females typically earn less. For full-time female graduates, the maximum total repayment is £75 786 for any initial debt above £45 988. For full-time male graduates, this maximum repayment is £110 644 for any initial debt above £65 145, again as any debt is written off after 30 years. As demonstrated by the ‘crossover point’ in figure 4, for an initial debt below almost exactly £50 000, female graduates repay more despite earning less because their debt lasts longer and accrues more interest charges. For graduates with initial debt above £50 000, male graduates typically repay more because their average yearly salaries are higher.

Repayment models were also created to include both part-time and full-time medical graduates, as some graduates will have periods of part-time work during their careers (see online supplementary figures S2 and S3). As female graduates now have even lower average yearly salaries, the maximum total repayment falls to £61 410, and therefore more debt is written off in the 30th year. For very low levels of initial debt, female graduates who earn less may repay more compared to higher earning females as they accrue more interest charges. This is less obvious for male graduates due to the low proportion of part-time workers in this group.

## Discussion

This is the first analysis concentrating on UK LFS respondents with a medical degree. It confirms that medical graduate salaries are correlated with gender. This gender difference has an impact on the repayment of potential graduate debt. For those graduates who are able to repay the debt in the 30-year period, a higher yearly salary is advantageous as early repayment means fewer interest charges are accrued. Curiously, for those who do not repay their total debt within the 30-year period, as is the case for the average female medical graduates, a lower salary means they repay less because yearly repayments are lower and the residual debt is written off.

While the overall LFS response rate is 50–70%, the specific response rate for medical graduates is not known.^11^ Though it is unclear if the LFS represents a true cross section of the 250 000 doctors registered with the General Medical Council (GMC), the observed rise in the number of female medical graduates within the LFS is similar to that seen by official GMC statistics.^17^ This is despite a constant 51.5% of females in the entire LFS sample. In addition, the proportions of LFS respondents working in hospital and non-hospital settings are similar to those reported by the GMC specialist and general practice (ie, family doctor) registers. Taken together, this suggests that the sampling of medical graduates by the ONS is representative.

Assumptions were made in the construction of the model and projected salaries. These calculations focused on the patterns of debt repayment for a medical graduate on an average salary. The impact of voluntary early repayments or non-SLC bank loans were not analysed here, but it has been estimated that non-SLC loans (eg, credit cards, overdraft and bank loans) constitute only 17% of the overall debt for individual medical graduates.^2^ It is unclear if this is the case for all medical graduates or whether this is limited to certain groups, for example, graduates from lower socioeconomic backgrounds. The models in this analysis assumed that medical graduate salaries will rise in line with inflation as they have done between 1999 and 2014 (see online supplementary figure S1) Finally, it should be noted that this study investigated the salaries and repayments of debt for medical graduates and no attempt was made to analyse the costs of failing to complete a degree or estimate the net returns of investing in a medical degree.

The disparity between male and female salaries is well documented across different careers and countries^18^ ^19^ and similar patterns have been documented in medicine.^7^ ^20^ ^21^ Reports based on non-selected samples of less than a thousand UK medical respondents again suggest that gender differences in salaries exist despite rigid pay scales.^7^ In the LFS data presented here, this gender gap in salaries begins at age 30, widens subsequently and is largely the result of a significant hourly wage difference between the genders. A range of factors have been cited as an explanation for these gender-wage differences which not only include discrimination but also career breaks, career choices, part-time work and motherhood.^22^ ^23^

University tuition fees have risen since they were introduced in 1998, as has graduate debt. Prior to the substantial 2012 rise in tuition fees, the Push National Debt Survey 2011 calculated average yearly student debt at £5681 from a poll of over 2000 university students in England.^21^ The BMA has estimated that medical graduates could owe £70 000 in loans despite NHS bursaries. The present calculations suggest that the typical debt for medical students living away from home could be nearly £82 000 at 2014 prices when one includes the projected interest charges accrued during the time at university.

The National Audit Office^24^ (NAO) presented a single simulation for a doctor graduating with an initial debt of £71 000, suggesting that they would clear the debt 25 years after graduating. However, in the simulations presented here, neither a male nor a female medical graduate with an initial debt of £70 022 (see column 9 in online supplementary tables S2 and S3) would ever repay the debt before it is written off. This difference is because the NAO analysis is based on salaries from doctors’ pay scales while our analysis is based on survey data on the earnings of all medical graduates.

Furthermore, gender differences in medical salaries highlight an inconsistent relationship between salaries and total repayments of debt. The complexities of the SLC loan contract mean that the effects on total repayments of lower female salaries vary with the level of initial graduate debt. For low levels of initial graduate debt, females will typically end up repaying more than males because their debt accrues more interest charges. For high levels of initial graduate debt, females could end up repaying less because their salaries are typically lower and the debt is written off after the 30th year. This possibility of an inverse relationship between lifetime repayments and earnings, at higher levels of earnings, does not seem to have been documented elsewhere.

Student debt is a particularly emotive issue in many countries and has been widely discussed in the UK press^25^ ^26^ and academic research.^27^ ^28^ It seems reasonable that these repayment variations may actually exist across many graduate careers in the UK. It is also apparent that at the current level of fees, even small changes in the student loan contract will have substantial implications for lifetime wealth across different income groups, across male and female graduates, and on the sustainability of the student loans system.
